# How Do Surface Polar Molecules Contribute to High Open‐Circuit Voltage in Perovskite Solar Cells?

**DOI:** 10.1002/advs.202205072

**Published:** 2023-04-20

**Authors:** Yinyi Ma, Chengsong Zeng, Peng Zeng, Yuchao Hu, Faming Li, Zhonghao Zheng, Minchao Qin, Xinhui Lu, Mingzhen Liu

**Affiliations:** ^1^ School of Materials and Energy University of Electronic Science and Technology of China Chengdu 611731 P. R. China; ^2^ Department of Physics The Chinese University of Hong Kong Shatin Hong Kong SAR 999077 China; ^3^ State Key Laboratory Electronic Thin Film and Integrated Devices University of Science and Technology of China Chengdu 611731 P. R. China

**Keywords:** dipole, field effect passivation, open‐circuit voltage, surface modification

## Abstract

To date, the improvement of open‐circuit voltage (*V*_OC_) offers a breakthrough for the performance of perovskite solar cells (PSCs) toward their theoretical limit. Surface modification through organic ammonium halide salts (e.g., phenethylammonium ions PEA^+^ and phenmethylammonium ions PMA^+^) is one of the most straightforward strategies to suppress defect density, thereby leading to improved *V*_OC_. However, the mechanism underlying the high voltage remains unclear. Here, polar molecular PMA^+^ is applied at the interface between perovskite and hole transporting layer and a remarkably high *V*_OC_ of 1.175 V is obtained which corresponds to an increase of over 100 mV in comparison to the control device. It is revealed that the equivalent passivation effect of surface dipole effectively improves the splitting of the hole quasi‐Fermi level. Ultimately the combined effect of defect suppression and surface dipole equivalent passivation effect leads to an overall increase in significantly enhanced *V*_OC_. The resulted PSCs device reaches an efficiency of up to 24.10%. Contributions are identified here by the surface polar molecules to the high *V*_OC_ in PSCs. A fundamental mechanism is suggested by use of polar molecules which enables further high voltage, leading ways to highly efficient perovskite‐based solar cells.

## Graphical abstract

A passivation strategy using polar molecular phenmethylammonium iodide (PMAI) is introduced to enhance performance of FA‐MA hybrid (FA: HC(NH_2_)_2_; MA: CH_3_NH_3_) perovskite solar cells. The dipole‐induced equivalent passivation effect is revealed successfully by PMAI and an applicable role of polar molecular passivators is declared. Overall, combining the molecular‐bonding defect passivation effect, the PMAI leads to a tremendous increase over 100 mV in *V*_OC_.

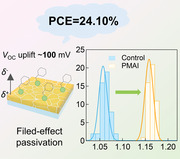

## 1 Introduction

The efficiency of hybrid organic–inorganic perovskite solar cells (PSCs) has currently rocketed to 25.7%^[^ ^1^ ^]^ since its first launch in 2009.^[^ ^2^ ^]^ Substantial effort has been carried out to push the device performance to its theoretical limit.^[^ ^3^ ^]^ The current record of short‐circuit current *J*_SC_ of 26.5 mA cm^−2[^ ^4^ ^]^ and fill factor of 86%^[^ ^5^ ^]^ have been demonstrated, which almost reached the theoretical limit for single‐junction PSCs, respectively. However, there still remains room for improvement in the open‐circuit voltage (*V*_OC_), which becomes a crucial role for further leaps in the power conversion efficiency (PCE) of PSCs.^[^ ^6^ ^]^ It is commonly considered that quasi‐Fermi level splitting of the perovskite layer dominates the output voltage of devices.^[^ ^7^ ^]^ Whereas the surface defects usually lead to fast recombination of charge carriers,^[^ ^8^ ^]^ by which efficient Fermi level splitting is unattainable. Therefore, by suppressing defect‐induced nonradiative recombination and maintaining high carrier density it has been a mainstream to improve the device *V*_OC_ in PSCs communities.

One straightforward approach is to passivate the surface defects.^[^ ^9^ ^]^ Effective bonding between additional molecule groups and defect sites helps to maintain sufficient photoinduced carrier densities leading to minimized voltage losses. To date a large number of different surface passivation techniques including film surface formation during fabrication processes,^[^ ^10^ ^]^ post‐treatment on top of perovskite films,^[^ ^11^ ^]^ and interfacial engineering between the perovskite and charge transport layer^[^ ^12^ ^]^ have been widely studied. In recent years, deposition of organic functional molecules onto the perovskite layer in devices has become the long‐lasting focus in PSCs communities.^[^ ^9b^ ^]^ Among them, phenylalkylamine salt is one of the most popular passivators to enhance device performance.^[^ ^13^ ^]^ For example, phenmethylammonium bromide (PMABr) has been proposed to modify the surface morphology of perovskite by forming microstructures at the interface while leading to an improvement in efficiency and stability.^[^ ^14^ ^]^ Zhu et al. employed phenmethylammonium chloride (PMACl) as a passivation agent to modify the surface of perovskite, which greatly alleviated the nonradiative recombination effect. The fabricated tandem device with all‐inorganic perovskite and organic absorbers as top and bottom sub cells, respectively, achieves a remarkably high PCE of 18.06%.^[^ ^15^ ^]^ In addition, phenethylammonium iodide (PEAI) also effectively reduces defect‐states and suppresses nonradiative recombination in PSCs with a certified PCE of 23.3%.^[^ ^9b^ ^]^

On the other hand, the introduction of external electric‐functional layers between perovskite and carrier transport layers also provides modulation of surface carrier densities, through the so‐called electric field‐effect passivation (FEP) effect, which has been widely applied in silicon photovoltaics techniques.^[^ ^16^ ^]^ External electric fields induced by aligned surface dipole layers at the interface may boost the built‐in electric field at the interface,^[^ ^17^ ^]^ which separates electrons and holes and thus suppresses carrier recombination, being equivalent to the molecular passivation effect from the passivation perspective.^[^ ^18^ ^]^ The FEP has been recently proposed in the optimization of PSCs devices, by adding a dipolar MoO*_x_* layer between the perovskite and hole‐transporting layer. It significantly resulted in an equivalent molecular passivation effect and thus enhanced *V*_OC_.^[^ ^19^ ^]^ Meanwhile, Ansari et al. reached a minimized voltage deficit of 0.37 V in a trication hybrid perovskite system by depositing an additional layer of AzPbI_3_ onto the perovskite film, in which Az brought in the dipole moment effect and consequently shifted the work function.^[^ ^20^ ^]^ Moreover, by embedding organic dipolar molecules such as ammonium salts^[^ ^21^ ^]^ and thiaazulenic derivatives,^[^ ^22^ ^]^ it shows the formation of a self‐oriented dipolar layer and also leads to effective enhancement in the device *V*_OC_. By using common dipolar molecules with diverse passivation groups, it enables the feasible introduction of the dipolar layers.

One may naturally expect a synergic enhancement on *V*_OC_ by both passivation and dipole effect from the additive molecules. The idea indeed has been discussed in some reported passivation works.^[^ ^20^, ^23^ ^]^ However, bearing in mind that only oriented dipole moment contributes to *V*_OC_ enhancement, the dipole moment effect in enhancement in device performance is still in debate. Fundamentally, there is barely an in‐depth quantitative understanding of such enhancement mechanisms. There remains a myth of the dominant contribution of the dipolar molecules to the improved *V*_OC_ in PSCs devices. In this work, we applied the ionic molecule phenmethylammonium iodide (PMAI), which is the most commonly used organic ammonium iodide salt in surface passivation research.^[^ ^24^ ^]^ In particular, the PMA^+^ ions assemble upright‐oriented dipole moment at the perovskite layer surface, via molecular bonding to perovskite interface lattice. We found those surface dipoles induced an enhancement in the interfacial built‐in electric field. Through quantitative analysis of carrier densities, we revealed that the dipole‐induced FEP effect also plays a mainstay role for the overall increase in *V*_OC_ by increasing the hole quasi‐Fermi level splitting over 100 mV. The molecular bonding also jointly resulted in a noticeable molecular passivation effect which synergically contributed 40 mV to the enhancement in output voltage. We eventually realized a high *V*_OC_ over 1.175 V in a PMAI‐processed FA‐MA hybrid (FA: HC(NH_2_)_2_; MA: CH_3_NH_3_) perovskite device system and the corresponding device showed a champion PCE of 24.10%. Our work clarifies the contributions to *V*_OC_ by the surface dipoles, thus realizing a way in *V*_OC_ enhancement by adding polar molecule passivators.

## 2 Results and Discussion

**Figure 1:**
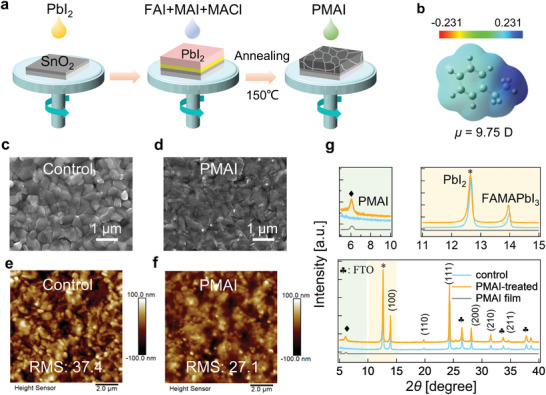
Preparation process and surface morphology. a) Schematic diagram of preparation processes of perovskite films. b) Calculated electronic density distributions with dipole moment of PMA^+^. c,d) Top‐view SEM morphology of perovskite film without and with PMAI treatment. e,f) The surface AFM images of the perovskite films without and with PMAI treatment. g) XRD patterns of control and PMAI‐treated samples compared with PMAI films. ♦, *, and ♣ represent PMAI, PbI_2_, and FTO, respectively.

The work was based on a hybrid FA_1−_ *_x_* MA*_x_* PbI_3_ perovskite system by a two‐step spin‐coating method. The synthesis process is schematically depicted in **Figure** **1**a. The PbI_2_ precursor was first spin‐coated on a SnO_2_ substrate. The perovskite layer was then formed by casting the mixed precursor including Formamidinium iodide (FAI), Methylammonium iodide (MAI), and MACl atop the PbI_2_ layer, followed by an annealing process at 150 °C. The PMAI dissolved in isopropanol/toluene (TL) mixed solvents was subsequently spin‐coated onto the perovskite surface without any further annealing process. Figure 1b shows the structure and electronic density distributions of PMA^+^, which presents a 9.75 Debye dipole moment at the optimized structure. Scanning electron microscopy images (SEMs) in Figure 1c,d clearly illustrate that the perovskite layer after PMAI treatment show appearance of additional skinning on top of the perovskite layer, which we assigned to the added PMAI salt.^[^ ^9b^ ^]^ In addition, the roughness of the perovskite layer has been reduced as shown in atomic force microscopy （AFM） (Figure 1e,f). In further, the effect of PMAI post‐treatment is considered marginal on the bandgap of the perovskite film (Figure S1, Supporting Information). Consistently, X‐ray diffraction (XRD) patterns (Figure 1g) indicate an additional diffraction peak at 6.1° in the presence of PMAI, which is in line with the PMAI‐only film diffraction pattern. Beyond above, identical perovskite lattice phases were observed in both samples and no additional XRD peaks of low‐dimensional perovskite structures were found. The effects of post‐treatment with different PMAI concentrations were further examined and shown in Figure S2 in the Supporting Information. With increasing ammonium salt concentration, only the diffraction peak of the PMAI salt alone was reinforced.

**Figure 2:**
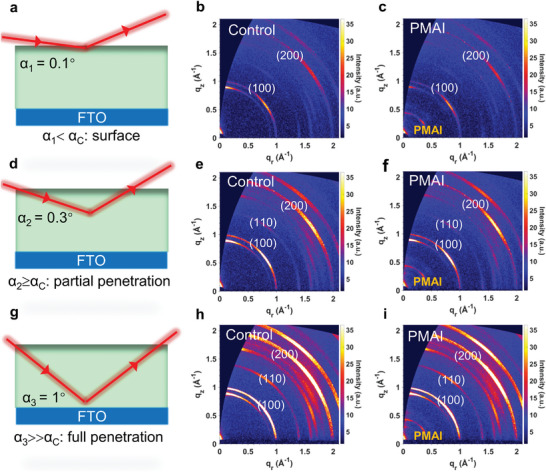
The grazing‐incidence wide‐angle X‐ray scattering (GIWAXS) images of perovskite films. a,d,g) Schematic diagram of detection depth at different incident angles, *α*_c_ is the critical angle. The GIWAXS patterns of b) Control film and c) PMAI‐treated film at an incident angle of 0.1°. The GIWAXS patterns of e) Control film and f) PMAI‐treated film at an incident angle of 0.3°. The GIWAXS patterns of h) Control film and i) PMAI‐treated film at an incident angle of 1°.

Grazing‐incidence wide‐angle X‐ray scattering (GIWAXS) was used to further characterize the crystal structure of the films before and after PMAI treatment (**Figure** **2**). Adjusting the incidence angle of the incident beam to control the X‐ray penetration depth allows us to easily investigate the depth profiles of the crystal structure within the film using GIWAXS measurements.^[^ ^25^ ^]^ When the incidence angle is 0.1° (Figure 2a), only structural information on the very surface of the film can be detected due to total X‐ray reflection. For an incident angle of 0.3° (Figure 2d), the obtained GIWAXS patterns reflect the bulk layer information of the perovskite film. For an incident angle of 1° (Figure 2g), the full penetration of X‐ray guarantees the detection of the structural information throughout the whole film.^[^ ^26^ ^]^ Compared with the control sample, a significant scattering peak of the PMAI salt at *q* = 0.437 Å^−1^ is observed in the GIWAXS patterns with an incident angle of 0.1° (Figure 2b,c), which is consistent with the coplanar XRD results (Figure 1g). The corresponding intensity profiles are shown in Figure S3a,b in the Supporting Information. It is worth noting that, as the X‐ray penetration depth increases, the scattering signals from PbI_2_ and perovskites are gradually enhanced whereas the peak intensity of PMAI remains almost the same, indicating that PMAI mostly reserved at the surface of the perovskite film (Figure S3c, Supporting Information). The intensity ratio of the PbI_2_/perovskite (100) phase was calculated from the polar intensity distribution, and the PMAI‐treated films all showed fewer PbI_2_ residues (Figure S3d, Supporting Information). In the meantime, two indistinct additional peaks located at *q* = 0.306 and 0.554 Å^−1^ emerge in the GIWAXS patterns with higher incident angles (Figure 2e,f,h,i), implying the formation of a low‐dimensional perovskite phase is only limited, as expected. Besides, the GIWAXS polar intensity profiles at different incident angles are summarized in Figure S4a,b in the Supporting Information to evaluate the perovskite crystal orientation. Both perovskite films present a preferred orientation at 55° while the film with PMAI shows a better orientation due to the smaller full width at half maximum (FWHM) value (Figure S4c, Supporting Information).

**Figure 3:**
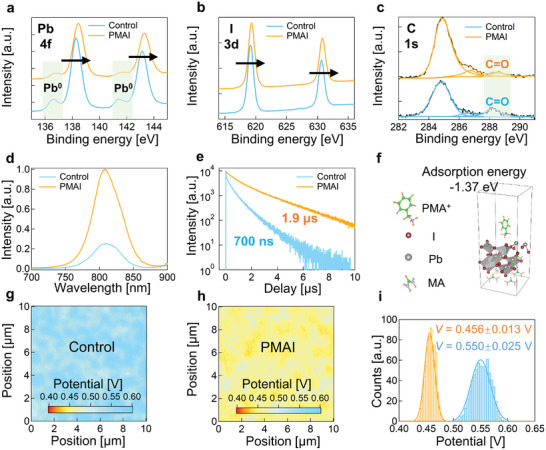
Characterizations of surface bonding and passivation effect. a) XPS spectra of Pb 4f. b) XPS spectra of I 3d. c) XPS spectra of C 1s. d) Steady‐state PL of the perovskite films without and with PMAI treatment. e) Time‐resolved PL of the perovskite films upon excitation at 510 nm. f) The calculated adsorption energy of perovskite and PMA^+^. g) Contact potential difference distribution for the control sample. h) Contact potential difference distribution for PMAI‐treated sample. i) Histograms of contact potential difference for control (blue) and PMAI‐treated (orange) films.

The PMA^+^ ion contains an ammonium group, which enables bondings with under‐coordinated I^−^ or Pb^2+^, resulting in a potential passivation effect upon the perovskite surface. In addition, I^−^ ions also possibly passivate vacancy defects at grain boundaries as schematically illustrated in Figure S5 in the Supporting Information.^[^ ^27^ ^]^ We applied X‐ray photoelectron spectroscopy (XPS) to reveal existing molecular bonding and chemical interactions between the perovskite and PMAI. Figure S6b in the Supporting Information shows that a shoulder peak emerges at a binding energy of 402 eV in the presence of PMAI.^[^ ^28^ ^]^ It is assigned to the typical N 1s orbital in PMA^+^, thus confirming the existence of PMA^+^ at the perovskite surface. Moreover, we observed an energy shift of both Pb 4f and I 3d peaks in **Figure** **3**a,b respectively toward higher binding energies for the PMAI processed perovskite film, in comparison to that of the control one. This shift is attributed to the bonding between PMA^+^ and I^−^/Pb^2+^. In particular, as shown in Figure 3a, the peak of lead Pb^0^ is obviously decreased by PMAI, indicative of effective suppression of Pb^0^ deep‐level defects which are possible recombination centers leading to deteriorating device efficiency.^[^ ^29^ ^]^ Interestingly, the C=O peak (288.2 eV) originating from external oxygen/moisture was significantly suppressed after PMAI treatment (Figure 3c), suggesting that the hydrophobic benzene ring structure is propitious for mitigating the degradation of perovskite.^[^ ^9b^ ^]^ Photoluminescence (PL) spectroscopy measurements (Figure 3d,e) reveal remarkably high PL emission intensity and a prolonged PL lifetime from 700 ns to 1.9 µs of perovskite films with the addition of PMAI (Table S1, Supporting Information), as a direct consequence of the interfacial passivation effect. Density functional theory (DFT) simulations of density of states (DOS) also exhibit obvious elimination of trap states under the passivation effect of PMA^+^ (see Figure S7, Supporting Information). In addition, first‐principle simulation proves effective bonding between PMA^+^ and perovskite lattice with a notable adsorption energy of −1.37 eV. Furthermore, this simulation suggests the most likely molecular configuration with minimum Gibbs energy, in which PMA^+^ moieties are spontaneously upright oriented with positive charges pointing toward the perovskite (as shown in Figure 3f). In fact, this self‐oriented configuration may take advantages of the short branched chain, which enables less free pitching of the negative‐charged benzene ring.

To observe the vertical orientation of the bonding between PMAI and the perovskite surface, we measured the workfunction of the perovskite layer using calibrated Kelvin probe force microscopy with the architecture displayed in Figure S8 in the Supporting Information. The workfunction of the microscopy probe was calibrated using a standard Au film substrate (see Figure S9, Supporting Information). We measured the workfunction of the perovskite film surface on SnO_2_/fluorine‐tin‐oxide (FTO)/glass substrate and all measurements were conducted under illumination conditions using a fiber halogen light source (Figure 3g,h). Compared to Control films, the surface potential distribution of the PMAI treated perovskite film is more uniform, which assists in minimizing the compounding within the device. The workfunction distributions are shown in Figure 3i. PMAI‐treated film exhibits a significantly enhanced workfunction of 4.54 eV in comparison to that of 4.44 eV in perovskite film without PMAI. This indicates the presence of an interfacial dipole with a positive charge pointing to the active layer.^[^ ^30^ ^]^ The increase of 100 meV in workfunction indicates an enhanced hole extraction ability (in contrast to electron extraction), which strongly suggests that the *V*_OC_ loss caused by the PMAI interface dipole layer is correspondingly suppressed by more than 100 mV.

**Figure 4:**
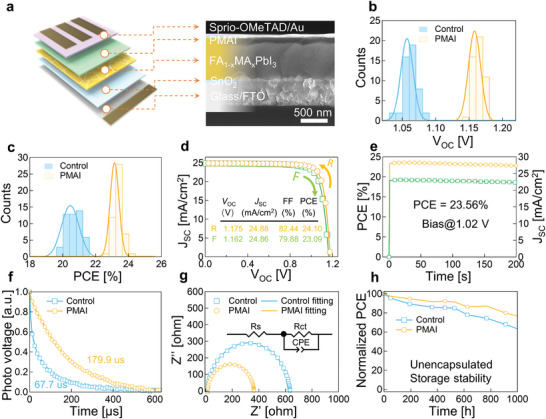
Device structure and photovoltaic performance. a) Device structure of perovskite solar cells. b,c) Distribution of voltage and efficiency. d) *J–V* curves measured by reverse and forward scans of the champion cell with active area of 0.072 cm^2^. e) Steady‐state PCE and current density of the champion device. f) Normalized transient photovoltage decay curves for the solar cells. g) Electrochemical impedance spectroscopy measurements. h) Long‐term stability of unencapsulated devices without and with PMAI treatment (humidity: 30% ± 5%).

We fabricated solar cell devices based on a planar heterojunction structure FTO/SnO_2_/FA_1−_ *_x_* MA*_x_* PbI_3_/PMAI/Spiro‐OMeTAD/Au, as shown in **Figure** **4**a. SEM images indicate a thickness of 800 nm for the perovskite layer. We achieved a remarkable increase of the average *V*_OC_ from 1.06 to 1.16 V in PSCs after the PMAI treatment on the perovskite film, corresponding to an enhancement of average PCEs from 20.70% to 23.4% (Figure 4b,c). It is noteworthy that there is almost no change in *J*_SC_ compared to the control device (Figure S10, Supporting Information). After optimization, the PMAI‐processed device reaches a champion PCE of 24.10% corresponding to a high *V*_OC_ of 1.175 V, as well as a short‐circuit current density *J*_SC_ of 24.88 mA cm^−2^ and a fill factor over 82.44% (Figure 4d). The integrated current density from the external quantum efficiency measurements was 24.69 mA cm^−2^ (Figure S11, Supporting Information), in good agreement with that measured by the solar simulator. The steady output for the champion device shows a quasi‐steady output of 23.56% at the maximum power output point upon a constant bias at 1.02 V (Figure 4e).

We further systematically characterized the optoelectronic properties of the PMAI processed devices. Figure 4f shows that the photovoltage decay lifetime of PMAI‐processed devices increases to 179.9 µs, indicating less overall Shockley–Read–Hall recombination rates that may be attributed to suppressed surface recombination process by PMA^+^. Electrochemical impedance spectroscopy (EIS) measurements were also conducted to compare the electronic characteristics of the device with and without PMAI (Figure 4g). At a bias of 1.0 V under dark conditions, equivalent circuit model (Table S2, Supporting Information) shows that PMAI devices possess a smaller charge transfer resistance (*R*_ct_) in the high‐frequency region, suggesting an efficient charge transfer process leading to improved fill factor as observed in the resulted devices. Additionally, the enhanced storage stability of our PMAI‐treated devices without unencapsulation under ambient conditions (Figure 4h) and operational stability under continuous light illumination (Figure S12, Supporting Information) were also demonstrated. This may be due to the hydrophobicity of the benzene ring in PMA^+^.

**Figure 5:**
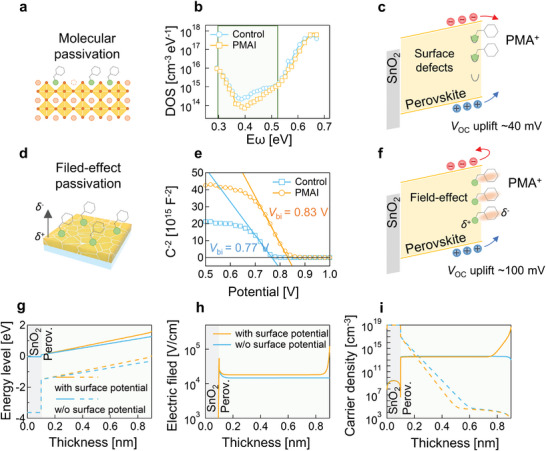
Field‐effect passivation and carrier density simulation. a) PMA^+^ bounded with Pb dangling defects at the perovskite surface. b) Trap state density by thermal admittance spectroscopy. c) *V*_OC_ uplift by surface defects passivation. d) Schematic diagram of field‐effect passivation. e) Mott–Schottky analysis of perovskite solar cells at 1 kHz. f) *V*_OC_ uplift by Field‐effect passivation. g) Energy level, h) electric field, and i) carrier density distribution along thickness of SnO_2_/perovskite films by wx‐AMPS simulations.

We carry out quantitative analysis in order to distinguish contributions to the *V*_OC_ enhancement. We first attempt to estimate the voltage enhancement by the defect passivation effect (**Figure** **5**a), by considering changes in surface defect density. Thermal admittance spectroscopy (TAS) measurements were carried out and it enables resolving the energy‐dependent distribution for densities of the defects in perovskite films (Figure 5b). Details of TAS measurements are provided in the Supporting Information. In comparison to the control device, the PMAI‐processed one exhibited obviously decreased densities of defect states. Bearing in mind that only the film surface was processed by PMAI with no further treatment such as annealing, the overall decrease in defect states is likely due to the change in surface defects. Here the change of TAS may reasonably reflect the changes of the surface defect density as we rationally assumed only the surface of the film was modified by PMAI. Additionally, the defect states within a range between 0.3 and 0.52 eV are commonly assigned to the surface defects.^[^ ^31^ ^]^ Overall, according to the TAS results, the change of the surface defects due to the passivation effect predicts an increase of the carrier density of 6 × 10^13^ cm^−3^ after PMAI treatment.^[^ ^32^ ^]^ In fact, a rational approximation of the increase of 5 × 10^15^ cm^−3^ in surface carrier density by assuming a trap‐active layer thickness of 10 nm. We further estimated the maximum *V*_OC_ through the obtainable quasi‐Fermi level splitting at surface^[^ ^33^ ^]^ and thus calculated the change of the output voltage with a straightforward increase, *∆V*_OC_ = 40 mV (Figure 5c) (calculation details in the Supporting Information). Obviously, the increment by the defect‐bonding passivation effect does not overwhelmingly count for the overall enhancement of *∆V*_OC_ by over 100 mV.

Therefore, the dipole‐induced surface potential must be included PMA^+^ moieties are bounded with the dangling Pb defects at the perovskite surface, as revealed by XPS measurements. According to the molecular configuration, self‐oriented upward dipoles were expected. The configuration of PMA^+^ moieties aligned a dipole direction along the built‐in electric field at the interface between the perovskite layer and hole transporting layer (HTL) (Figure 5d), leading to an enhancement in the built‐in electric field. The built‐in potential (*V*_bi_) difference between the PMAI‐treated and control samples is supported by the Mott–Schottky data.^[^ ^34^ ^]^ Figure 5e shows the typical depletion layer behavior (*C*_dl_) with negligible low‐frequency excess capacitance (*C*_s_). The *V*_bi_ of the PMAI‐treated sample increases by 60 mV compared to the control sample. It should be noted that the increase in *V*_bi_ is an evident result of the higher *V*_OC_ from the PMAI‐treated device.

An intuitive interpretation of the enhanced built‐in field effect is commonly given as follows. The electric field pointing from perovskite to HTL drives the holes and simultaneously repels the electrons back to the perovskite, thus less recombination occurs at the interface, resulting in an equivalent passivation effect of the surface defects (Figure 5f). We accordingly proposed a quantitative analysis of the field‐effect passivation effect brought by the dipole molecules. Through carrier density simulation by using the 1D device simulator wx‐AMPS, which gives solutions to the diffusion and recombination differential equations of carriers.^[^ ^35^ ^]^ We focused on the change of the hole density under the enhanced built‐in electric field at the perovskite interface. Details of simulation parameters are provided in Table S3 in the Supporting Information. An upward band bent was introduced to simulate the PMA^+^ dipole effect and consequently conduct an enhanced built‐in field. The simulation was based on a device structure of SnO_2_/perovskite. Figure 5g shows simulated energy band alignments under Air Mass （AM） 1.5 full illuminations. The additional bent band energy was set to 50 meV. It obviously elevated the built‐in electric field at the interface as shown in Figure 5h. The changes resulted in a significant increase in hole density at the interface, by comparison to the perovskite film with no surface potential (Figure 5i). The simulation clearly reveals the suppression effect upon carrier recombination from the enhanced built‐in field.

We thus investigated the dipole effect in terms of the equivalent passivation effect by the field‐effect. We again apply the simple approximation in order to quantify the increase in *V*_OC_ with the increasing hole density, from the perspective of quasi‐Fermi level splitting. The voltage loss, *V*_loss_ caused by hole density is *V*_loss_ = *kT* · ln(*p*/*N*_V_) according to Equation (S3) in the Supporting Information. With an estimation of three‐order higher hole density at the interface from the device simulation, we found a decrease over 100 mV in *V*_loss_ as a result of the increase in hole density. Together with the contribution from the molecular passivation, the total uplift in *V*_OC_ is in line with the observed increase of 100 mV with the coverage of PMAI at the interface between the perovskite and the HTL. We therefore suggest that the dipole‐induced FEP is the dominant contribution to the observed 100 mV increase in *V*_OC_ in devices.

So far we have established the mechanism of the increase in *V*_OC_ using PMAI. The *V*_OC_ enhancement was essentially attributed to the increased‐hole‐density induced quasi‐Fermi level splitting (QFLS) at the interface. We interpreted the change of the hole density from a perspective of passivation effect including molecular‐bonding and dipole field‐effect. The two ways were found to be jointly responsible for the 100 mV leap in *V*_OC_. In order to confirm the dipole‐induced FEP effect, we additionally deposited similar PEAI (phenylethylammonium iodide) molecules instead of PMAI onto the perovskite surface, by following Jiang et al.^[^ ^9b^ ^]^ Although the molecular dipole moment of PEA^+^ is more significant than PMA^+^ (see Figure S13, Supporting Information), simulations indicated a likely prone configuration of the benzene ring, as a result of the additional carbon in the branched chain which provides more flexible freedom of moiety pitching. Thus PEA^+^ may introduce less dipole‐induced FEP than PMA^+^. Consequently, PEAI‐treated devices showed a less significant *V*_OC_ increase than PMAI in Figure S14 in the Supporting Information. Our results are in line with reported *V*_OC_ improvement of 60 mV by PEAI in Jiang et al.’s work.^[^ ^9b^ ^]^ Finally, we extended this surface post‐treatment method to other types of devices. The results (FigureS15 and S16, Supporting Information) show that the *V*_OC_ enhancement is all significant.

## 3 Conclusions

In conclusion, we demonstrated that the surface‐treatment strategy based on an organic dipole layer PMAI effectively enhanced the performance of PSCs, corresponding to an impressively high *V*_OC_ over 1.175 V. Especially, we showed that the field‐effect passivation induced by the oriented PMA^+^ dipoles, can suppress carrier recombination leading to remarkably high hole density at the interface. We quantitatively suggested that the defect‐bonding and dipole field‐effect jointly contribute to such voltage improvement in devices. As a result, by working along both lines, the additional PMA^+^ lead to a great leap over 100 mV in *V*_OC_. Finally, the resulted PSCs devices exhibited a champion PCE of 24.10%, and improved long‐term stability under ambient conditions without encapsulation. Our findings provide an overall understanding of effects of polar molecules upon surface modification in perovskite devices and moreover suggest that the common surface passivation routes may be good enough to enable high *V*_OC_.

## 4 Experimental Section

### Materials

Materials in this work included FAI (99.9%, Dyesol), MAI (99.9%, Dyesol), lithium bis(trifluoromethanesulfonyl)imide (Li‐TFSI, 99.95%, Sigma‐Aldrich), SnO_2_ colloid precursor (Alfa Aesar, tin(IV) oxide, 15% in H_2_O colloidal dispersion), 4‐tertbutylpyridine (4‐tBP, 96%, Sigma‐Aldrich), chlorobenzene (CB, Sigma‐Aldrich), *N*,*N*‐dimethyiformamide (DMF, 99.8%, Sigma‐Aldrich), dimethylsulfoxide (DMSO, 99.5%, Sigma‐Aldrich), isopropanol (IPA,99.99%, Sigma‐Aldrich), toluene (TL,99.5%, Keshi), acetonitrile (ACN,99.8%, Sigma‐Aldrich), 4‐tertbutylpyridine (TBP,98%, Sigma‐Aldrich), Spiro‐OMeTAD (99.8%, Borun New Material Technology), PbI_2_ (99.99%, Sigma‐Aldrich). MACl (99.9%), and PMAI (99.5%) were purchased from Xi'an Polymer Light Technology in China. All chemicals were directly used without any further purification.

### Solar Cell Fabrication

FTO glass (≈8 Ω sq^−1^) was cleaned by ultrasonic cleaner using acetone and ethanol sequentially. Before use, the FTO was cleaned with plasma for 20 min. Then SnO_2_ colloid solution diluted by water (1:5) was spin‐coated on the substrate at 3000 rpm for 30 s, annealing in ambient air at 160 °C for 40 min. The substrate was then cleaned with ultraviolet ozone for 10 min before preparation of perovskite films. 1.5 m PbI_2_ in DMF: DMSO (9:1; v/v) solvent was stirred at 70 °C overnight and then was spin‐coated on the SnO_2_ substrate at 1500 rpm for 30 s, followed by an annealing process at 70 °C for 1 min. After cooling down, the FAI/MAI/MACl (90 mg: 6.3 mg: 9 mg in 1 mL IPA) solution was subsequently spin‐coated on the PbI_2_ layer at 2000 rpm for 30 s. Then, the film was annealed at 150 °C for 15 min in ambient air conditions (25–35% humidity). After the formation of the perovskite layer, different concentrations of PMAI solution dissolved in IPA: TL (5:5; v/v) was spin‐coated on the perovskite surface at 5000 rpm for 30 s. Finally, the hole‐transport layer was deposited by spin‐coating the Spiro‐OMeTAD solution at 3000 rpm for 30 s, which consisted of 72.3 mg Spiro‐OMeTAD, 17.5 µL Li‐TFSI solution (520 mg Li‐TFSI in 1 mL ACN) and 28.8 µL 4‐TBP in 1 mL CB. At last, a 100 nm Au electrode was deposited by thermal evaporation on top of the Spiro‐OMeTAD layer.

### MAPbI_3_ Preparation

1.3 m PbI_2_ in DMF: DMSO (9:1; v/v) solvent was stirred at 70 °C overnight and then was spin‐coated on the SnO_2_ substrate at 2300 rpm for 30 s, followed by an annealing process at 70 °C for 1 min. After cooling down, the MAI/MACl (60.4 mg: 4.7 mg in 1 mL IPA) solution was subsequently spin‐coated on the PbI_2_ layer at 1500 rpm for 30 s. Then, the film was annealed at 150 °C for 15 min in ambient air conditions (25–35% humidity).

### FACsPbI_3_ Preparation

The perovskite precursor solution was prepared by dissolving PbI_2_ (1.3 mmol), FAI (1 mmol), CsCl (0.2 mmol), and 0.49 mmol FACl additive in 1 mL of DMF/DMSO (4:1; v/v).The perovskite solutions were deposited by spin‐coating in a two‐step program at 1000 rpm for 10 s and 5000 rpm for 25 s. In the second step, 200 µL of CB was dropped on the top of the spinning film 10 s prior to the end of the program. After deposition, the film was annealed at 150 °C for 30 min on a hot plate in filled N_2_ glove box.

### Measurements and Characterization

Bruker D8 Advance diffractometer with Cu *K*_*α*_ radiation (*λ* = 1.5418 Å) and LYNXEYE_XE detector was used to record X‐ray diffraction patterns of films and powders. GIWAXS measurements were conducted at a Xeuss 2.0 small‐angle X‐ray scattering (SAXS)/wide‐angle X‐ray scattering (WAXS) laboratory beamline equipped with a Cu X‐ray source (8.05 keV, 1.54 Å) and a Pilatus3R 300 K detector. Three incident angles (0.1°, 0.3°, and 1°) were selected to represent the crystal structure of perovskite with different depths. Scanning electron microscope images were gained by FEI Inspect F50 electron microscope with electron energy of 10 keV. Steady‐state and time‐resolved PL decays were characterized by using FluoTime300 (PicoQuant). Delivered pulse energy was set to 35 pJ at 510 nm which corresponds to an excitation of carrier density on the order of 10^16^ cm^3^. The ultimate instrument response function was better than 25 ps for time‐resolved PL measurements. The repetition rate of laser diode was set to be 103 226 and 45 007 Hz, respectively. For PL measurements, the background subtractions were performed by multiexponential fittings with a constant term. XPS was detected by the Thermo Fisher Scientific Escalab 250Xi system by using a He discharge lamp (21.22 eV). The XPS spectra were calibrated using inorganic carbon at 284.8 eV. The *J–V* curves were measured by Keithley 2400 digital source‐meter and the devices were placed under simulated AM 1.5G irradiation (100 mW cm^−2^, xenon lamp, Newport). The effective area of the cell was defined by a metal mask (0.072 cm^2^). The electrochemical impedance spectra (EIS) of the solar cells were obtained by an IM6e Electrochemical Workstation (Zennium Pro). Mott–Schottky analysis of the data was measured at a frequency of 1 kHz with bias potentials from 0 to 1 V. The TAS was derived from the frequency‐dependent capacitance and voltage dependent capacitance. The KPFM measurements were conducted by an AFM (KEYSIGHT Technologies 7500) with a Pt‐coated conductive cantilever probe (Bruker, Model: SCM‐PIT‐V2). Transient photocurrent measurement was performed with a system excited by a 532 nm (1000 Hz, 3.2 ns) pulse laser. Transient photo voltage measurement was performed with the same system excited by a 405 nm Continuous‐wave (CW) laser (MDL‐III‐405). A digital oscilloscope (Tektronix, MSO5204B) was used to record the photocurrent or photovoltage decay process with a sampling resistor of 50 Ω or 1 MΩ, respectively.

### DFT Simulation

For PMA^+^ dipole moment, the ground state structure of the PMA^+^ cation were optimized by using DFT method,^[^ ^36^ ^]^ including Beckethree–Lee–Yang–Parr functional at 6‐31+G basis set.^[^ ^37^ ^]^ The electronic structure calculations in this work were performed by using Gaussian 09 program package. For molecular adsorption model, the adsorption energy (*E*_ads_) of adsorbed PMA^+^ was defined as $E_{\mathrm{ads}}=E_{{\mathrm{PMA}}^{+}/\mathrm{surf}}\;-E_{\mathrm{surf}}-E_{{\mathrm{PMA}}^{+}\left(g\right)}$, where *E*_PMA+/surf_, *E*_surf_, and *E*_PMA+(g)_ are the energy of adsorbed PMA^+^ adsorbed on the surface, the energy of clean surface, and the energy of isolated PMA^+^ molecule in a cubic periodic box. Vienna Ab Initio Package^[^ ^38^ ^]^ was employed to perform the DFT calculations within the generalized gradient approximation using the Perdew‐Burke‐Ernzerhof (PBE)^[^ ^39^ ^]^ formulation. The projected augmented wave potentials^[^ ^40^ ^]^ were used to describe the ionic cores and take valence electrons into account. The DOS of each model was calculated by the screened hybrid functional of Heyd, Scuseria, and Ernzerhof (HSE06).^[^ ^41^ ^]^ The equilibrium lattice constant of cubic FAPbI_3_ unit cell was optimized, when using a 4 × 4 × 4 Monkhorst‐Pack *k*‐point grid for Brillouin zone sampling. The surface slab was constructed with an FAPbI_3_ (001) sheet model with *p* (2 × 2) periodicity in the *x* and *y* directions and two stoichiometric layers in the *z*‐direction separated by a vacuum layer.

### Device Simulation

Simulation of device performance including energy level, electric field, and carrier density was performed using wx‐AMPS solar simulator. The simulation device structure was SnO_2_/perovskite. For SnO_2_, parameters were based on the defaults provided by the wx‐AMPS software and the thickness was set to be 100 nm. For the perovskite layer, the thickness was 800 nm determined by SEM measurements. The wavelength‐dependent absorption coefficients and bandgap were given by UV–vis measurements. Effective densities of valence *N*_V_ and conduction *N*_C_ band states were based on reported values of MAPbI_3_ and others were based on the previous work. Details are provided in the Supporting Information.

### Statistical Analysis

Statistical analysis was performed using Origin or Igor. 50 solar cells were used to generate the statistics shown in Figure 4b,c and fitted by a Gaussian function. The boxplot shows the statistical distribution of the device performance parameters, where the lower whisker, lower box edge, midline, upper box edge, and upper whisker refer to the minimum, 25th percentile, median, 75th percentile, and maximum value of the dataset, respectively. PCE data points for stability testing (including light soaking and ambient humidity aging), they were normalized to the PCEs measured before aging. Biexponential decay function was applied to the TRPL decay to infer the carrier extraction/recombination kinetics (Figure 3e). Linear fit applied to the Mott–Schottky plot (Figure 5e).

## Conflict of Interest

The authors declare no conflict of interest.

## Supporting information

Supporting Information

## Data Availability

The data that support the findings of this study are available from the corresponding author upon reasonable request.

## References

[1] NREL , Best Research‐Cell Efficiencies, https://www.nrel.gov/pv/cell‐efficiency.html (accessed: October 2022).

[2] A. Kojima , K. Teshima , Y. Shirai , T. Miyasaka , J. Am. Chem. Soc. 2009, 131, 6050. 19366264 10.1021/ja809598r

[3] a) M. Liu , M. B. Johnston , H. J. Snaith , Nature 2013, 501, 395; 24025775 10.1038/nature12509

[4] H. Min , M. Kim , S. U. Lee , H. Kim , G. Kim , K. Choi , J. H. Lee , S. I. Seok , Science 2019, 366, 749. 31699938 10.1126/science.aay7044

[5] W.‐Q. Wu , J.‐X. Zhong , J.‐F. Liao , C. Zhang , Y. Zhou , W. Feng , L. Ding , L. Wang , D.‐B. Kuang , Nano Energy 2020, 75, 104929.

[6] D. Guo , V. M. Caselli , E. M. Hutter , T. J. Savenije , ACS Energy Lett. 2019, 4, 855.

[7] P. Caprioglio , M. Stolterfoht , C. M. Wolff , T. Unold , B. Rech , S. Albrecht , D. Neher , Adv. Energy Mater. 2019, 9, 1901631.

[8] D. Luo , R. Su , W. Zhang , Q. Gong , R. Zhu , Nat. Rev. Mater. 2019, 5, 44.

[9] a) X. Zheng , B. Chen , J. Dai , Y. Fang , Y. Bai , Y. Lin , H. Wei , X. Zeng , J. Huang , Nat. Energy 2017, 2, 17102;

[10] a) M. Kim , G.‐H. Kim , T. K. Lee , I. W. Choi , H. W. Choi , Y. Jo , Y. J. Yoon , J. W. Kim , J. Lee , D. Huh , H. Lee , S. K. Kwak , J. Y. Kim , D. S. Kim , Joule 2019, 3, 2179;

[11] a) F. Gao , Y. Zhao , X. Zhang , J. You , Adv. Energy Mater. 2019, 10, 1902650;

[12] a) H. Min , D. Y. Lee , J. Kim , G. Kim , K. S. Lee , J. Kim , M. J. Paik , Y. K. Kim , K. S. Kim , M. G. Kim , T. J. Shin , S. Il Seok , Nature 2021, 598, 444; 34671136 10.1038/s41586-021-03964-8

[13] H.‐S. Yoo , N.‐G. Park , Sol. Energy Mater. Sol. Cells 2018, 179, 57.

[14] T. Bu , J. Li , Q. Lin , D. P. McMeekin , J. Sun , M. Wang , W. Chen , X. Wen , W. Mao , C. R. McNeill , W. Huang , X.‐L. Zhang , J. Zhong , Y.‐B. Cheng , U. Bach , F. Huang , Nano Energy 2020, 75, 104917.

[15] X. Wu , Y. Liu , F. Qi , F. Lin , H. Fu , K. Jiang , S. Wu , L. Bi , D. Wang , F. Xu , A. K. Y. Jen , Z. Zhu , J. Mater. Chem. A 2021, 9, 19778.

[16] M. Zhang , Q. Chen , R. Xue , Y. Zhan , C. Wang , J. Lai , J. Yang , H. Lin , J. Yao , Y. Li , L. Chen , Y. Li , Nat. Commun. 2019, 10, 4593. 31597916 10.1038/s41467-019-12613-8 PMC6785549

[17] a) J.‐H. Lee , J. Kim , G. Kim , D. Shin , S. Y. Jeong , J. Lee , S. Hong , J. W. Choi , C.‐L. Lee , H. Kim , Y. Yi , K. Lee , Energy Environ. Sci. 2018, 11, 1742;

[18] L. Liang , H. Luo , J. Hu , H. Li , P. Gao , Adv. Energy Mater. 2020, 10, 2000197.

[19] F. Wang , Y. Zhang , M. Yang , D. Han , L. Yang , L. Fan , Y. Sui , Y. Sun , X. Liu , X. Meng , J. Yang , Adv. Funct. Mater. 2020, 31, 2008052.

[20] F. Ansari , E. Shirzadi , M. Salavati‐Niasari , T. LaGrange , K. Nonomura , J. H. Yum , K. Sivula , S. M. Zakeeruddin , M. K. Nazeeruddin , M. Gratzel , P. J. Dyson , A. Hagfeldt , J. Am. Chem. Soc. 2020, 142, 11428. 32391696 10.1021/jacs.0c01704

[21] S. Yang , J. Dai , Z. Yu , Y. Shao , Y. Zhou , X. Xiao , X. C. Zeng , J. Huang , J. Am. Chem. Soc. 2019, 141, 5781. 30888171 10.1021/jacs.8b13091

[22] J. F. Butscher , S. Intorp , J. Kress , Q. An , Y. J. Hofstetter , N. Hippchen , F. Paulus , U. H. F. Bunz , N. Tessler , Y. Vaynzof , ACS Appl. Mater. Interfaces 2020, 12, 3572. 31799828 10.1021/acsami.9b18757

[23] F. Li , J. Yuan , X. Ling , L. Huang , N. Rujisamphan , Y. Li , L. Chi , W. Ma , ACS Appl. Mater. Interfaces 2018, 10, 42397. 30422618 10.1021/acsami.8b15870

[24] a) Z. Li , S. Wu , J. Zhang , K. C. Lee , H. Lei , F. Lin , Z. Wang , Z. Zhu , A. K. Y. Jen , Adv. Energy Mater. 2020, 10, 2000361;

[25] M. Qin , P. F. Chan , X. Lu , Adv. Mater. 2021, 33, 2105290. 10.1002/adma.202105290 34605066

[26] H. Zhang , M. Qin , Z. Chen , W. Yu , Z. Ren , K. Liu , J. Huang , Y. Zhang , Q. Liang , H. T. Chandran , P. W. K. Fong , Z. Zheng , X. Lu , G. Li , Adv. Mater. 2021, 33, 2100009. 10.1002/adma.202100009 33893688

[27] M. Abdi‐Jalebi , Z. Andaji‐Garmaroudi , S. Cacovich , C. Stavrakas , B. Philippe , J. M. Richter , M. Alsari , E. P. Booker , E. M. Hutter , A. J. Pearson , S. Lilliu , T. J. Savenije , H. Rensmo , G. Divitini , C. Ducati , R. H. Friend , S. D. Stranks , Nature 2018, 555, 497. 29565365 10.1038/nature25989

[28] F. Tan , M. I. Saidaminov , H. Tan , J. Z. Fan , Y. Wang , S. Yue , X. Wang , Z. Shen , S. Li , J. Kim , Y. Gao , G. Yue , R. Liu , Z. Huang , C. Dong , X. Hu , W. Zhang , Z. Wang , S. Qu , Z. Wang , E. H. Sargent , Adv. Funct. Mater. 2020, 30, 2005155.

[29] L. Wang , H. Zhou , J. Hu , B. Huang , M. Sun , B. Dong , G. Zheng , Y. Huang , Y. Chen , L. Li , Z. Xu , N. Li , Z. Liu , Q. Chen , L. D. Sun , C. H. Yan , Science 2019, 363, 265. 30655439 10.1126/science.aau5701

[30] Z. He , C. Zhong , X. Huang , W. Y. Wong , H. Wu , L. Chen , S. Su , Y. Cao , Adv. Mater. 2011, 23, 4636. 21905131 10.1002/adma.201103006

[31] N. Li , S. Tao , Y. Chen , X. Niu , C. K. Onwudinanti , C. Hu , Z. Qiu , Z. Xu , G. Zheng , L. Wang , Y. Zhang , L. Li , H. Liu , Y. Lun , J. Hong , X. Wang , Y. Liu , H. Xie , Y. Gao , Y. Bai , S. Yang , G. Brocks , Q. Chen , H. Zhou , Nat. Energy 2019, 4, 408.

[32] P. Zeng , Q. Zhang , Y. Zhang , B. Cai , G. Feng , Y. Wang , C. Zeng , W.‐H. Zhang , M. Liu , Appl. Phys. Lett. 2021, 119, 101101.

[33] T. Leijtens , G. E. Eperon , A. J. Barker , G. Grancini , W. Zhang , J. M. Ball , A. R. S. Kandada , H. J. Snaith , A. Petrozza , Energy Environ. Sci. 2016, 9, 3472.

[34] O. Almora , M. Garcia‐Batlle , G. Garcia‐Belmonte , J. Phys. Chem. Lett. 2019, 10, 3661. 31188609 10.1021/acs.jpclett.9b00601

[35] Y. Liu , Y. Sun , A. Rockett , Sol. Energy Mater. Sol. Cells 2012, 98, 124.

[36] P. Hohenberg , W. Kohn , Phys. Rev. 1964, 136, B864.

[37] C. Lee , W. Yang , R. G. Parr , Phys. Rev. B 1988, 37, 785. 10.1103/physrevb.37.785 9944570

[38] a) G. Kresse , J. Furthmuller , Phys. Rev. B 1996, 54, 11169; 10.1103/physrevb.54.11169 9984901

[39] J. P. Perdew , K. Burke , M. Ernzerhof , Phys. Rev. Lett. 1996, 77, 3865. 10062328 10.1103/PhysRevLett.77.3865

[40] a) G. Kresse , D. Joubert , Phys. Rev. B 1999, 59, 1758;

[41] A. D. Becke , J. Chem. Phys. 2014, 140, 18A301. 10.1063/1.4869598 24832308

