# Supplementary material for: How Do Surface Polar Molecules Contribute to High Open‐Circuit Voltage in Perovskite Solar Cells?
Source: Adv Sci (Weinh). 2023 Apr 20;10(17):2205072. doi: 10.1002/advs.202205072 (PMC10265051; doi:10.1002/advs.202205072)
Supplement: Supplementary file 1 — Supporting Information [file ADVS-10-2205072-s001.pdf]

ADVANCED SCIENCE
Open Access

Supporting Information

for *Adv. Sci.*, DOI 10.1002/advs.202205072

How Do Surface Polar Molecules Contribute to High Open-Circuit Voltage in Perovskite Solar Cells?

*Yinyi Ma, Chengsong Zeng, Peng Zeng, Yuchao Hu, Faming Li, Zhonghao Zheng, Minchao Qin, Xinhui Lu and Mingzhen Liu**

Supporting Information

How do Surface Polar Molecules Contribute to High Open-Circuit Voltage in Perovskite Solar Cells?

*Yinyi Ma [†], Chengsong Zeng [†], Peng Zeng, Yuchao Hu, Faming Li, Zhonghao Zheng, Minchao Qin, Xinhui Lu, and Mingzhen Liu**

Y. Ma, C. Zeng, Dr. P. Zeng, Dr. Y. Hu, Dr. F. Li, Z. Zheng, Prof. M. Liu
School of Materials and Energy, University of Electronic Science and Technology of China, Chengdu 611731, P.R. China.
E-mail: mingzhen.liu@uestc.edu.cn

Prof. M. Liu
State Key Laboratory Electronic Thin Film and Integrated Devices, University of Science and Technology of China, Chengdu 611731, China.
E-mail: mingzhen.liu@uestc.edu.cn

Dr. M. Qin, Prof. X. Lu
Department of Physics, The Chinese University of Hong Kong, Shatin 999077, Hong Kong SAR.

† These authors contributed equally to this work.

Thermal admittance spectroscopy (TAS)

TAS is a powerful technique for discovering the energy levels and the density distribution of trap states. The density of states can be derived from the angular frequency-dependent capacitance according to Equation:[1]

$$DOS(E_{\omega}) = -\frac{V_{bi}}{qW}\frac{dC}{d\omega}\frac{\omega}{K_{B}T} \qquad \text{Equation (S1)}$$

Where q is the elementary charge, C is the capacitance, ω is the applied angular frequency, K_B is the Boltzmann's constant and T is the temperature, respectively. V_{bi} and W are the built-in potential and depletion width which were gained from the Mott-Schottky analysis. The energy of the trap states energy level is defined by applied angular frequency ω:

$$E_{\omega} = K_{B}T ln\left(\frac{\omega_0}{\omega}\right) \qquad \text{Equation (S2)}$$

Where the ω_0 is the attempt to escape frequency. The trap states below the energy demarcation can capture or emit charges with the given ω and contribute to the capacitance.

Calculation Voc by quasi-Fermi level splitting

By following a rough approximation of the maximum V_{OC} through the obtainable quasi-Fermi level splitting at surface, we estimated the change of V_{OC} using the following equation:[2]

$$V_{\mathrm{OC}} = E_{\mathrm{F_n}} - E_{\mathrm{F_p}} = E_{\mathrm{G}} + kT \cdot ln\left(\frac{n}{N_{\mathrm{C}}}\right) + kT \cdot ln\left(\frac{p}{N_{\mathrm{V}}}\right) \qquad \text{Equation (S3)}$$

Where E_{Fn} and E_{Fp} are the quasi-Fermi levels for electrons and holes, respectively. n and p are the electron and hole density, while N_{C} and N_{V} are the effective density of states of the conduction and valence bands, respectively. N_{C} and N_{V} are determined by the intrinsic effective masses of electrons and holes. kT is the thermal energy and corresponds to 26 meV at room temperature.

The change of output voltage, ΔV_{OC} is thus given by $\Delta V_{OC} = kT \cdot ln(p/p_0)$ following an assumption of identical densities of carriers in conduction band and unchanged effective band state densities. p_0 and p are the valence band hole densities in the control and processed perovskite films, respectively. It is worth noting that p herein stands for the hole density at surface. We roughly approximate an initial photo-induced hole density p_0 in the pristine perovskite film based on the measured J_{SC} and PL lifetime τ, by $J_{SC} = p_0 d/\tau$ (d denotes the film thickness 800 nm herein). It predicts a hole density p_0 of 1.4×10^{15} cm^{-3} corresponding to J_{SC} of 24.84 $mAcm^{-2}$ (see Figure S17) and 0.7 μs PL lifetime in the control device. The increment in hole density thus leads to a straightforward increase, ΔV_{OC} = 40 mV. It is noted that the above approximation only gives an upper limit to ΔV_{OC}. Obviously, the increment by the defect-bonding passivation effect doesn't overwhelmingly count for the overall enhancement of ΔV_{OC} by over 100 mV.

Calculate the work function of the sample from the KPFM results

The KPFM technique is an electrical characterization method used to plot the surface potential or work function of a sample. The surface potential acquired from KPFM, termed as contact potential difference (CPD).The work function of the probe tip and the sample can be calculated using eq4[3]

$$\mathrm{CPD} = \frac{\Phi_{tip} - \Phi_{sample}}{e} \qquad \text{Equation (S4)}$$

CPD is the contact potential difference between the probe tip and the sample, Φ_{tip} and Φ_{sample} are the workfunction of the tip and the sample surface, respectively, and e is the elementary charge. Herein, the work function of the tip is calibrated using Au film (Φ_{Au} = 5.1 eV, CPD_{Au} = -106 mV). Therefore, the work function of the sample surface is calculated by eq5

$$\Phi_{\mathrm{sample}} = \Phi_{Au} + \mathrm{e}(\mathrm{CPD_{Au}} - \mathrm{CPD_{sample}}) \qquad \text{Equation (S5)}$$

Therefore, the workfunctions before and after PMAI treatment are 4.44 eV and 4.54 eV, respectively.

Figures and Tables

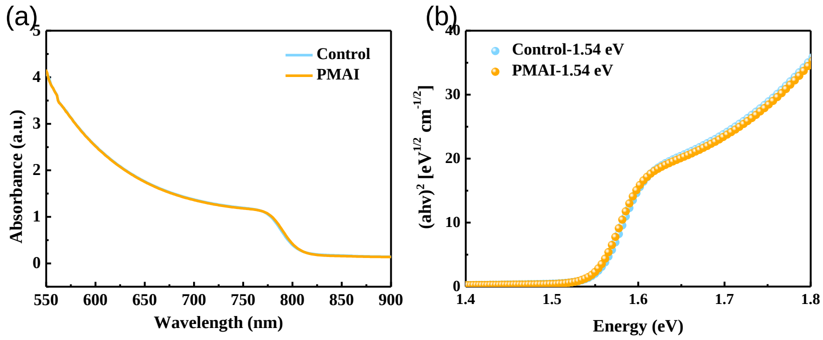

Figure S1. (a) UV-vis absorption spectra and the corresponding optical band gap (b) of the perovskite before and after PMAI treatment.

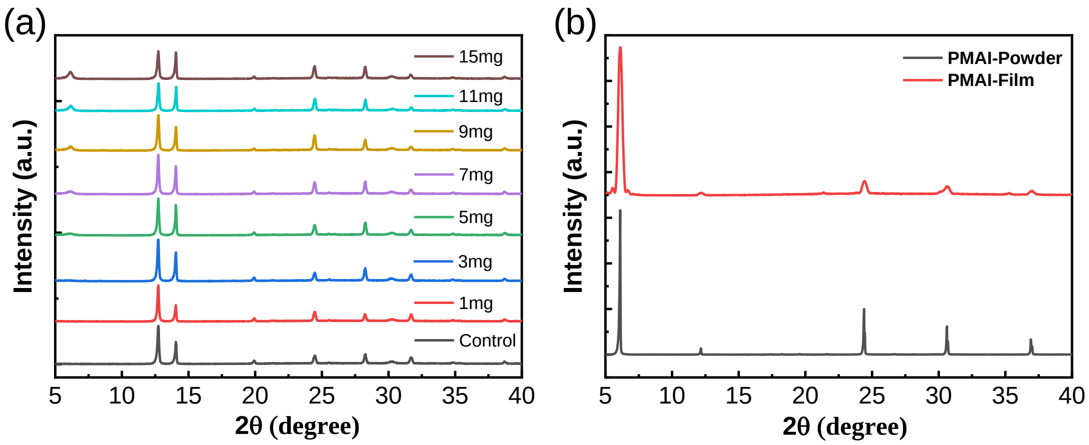

Figure S2. (a) XRD pattern of the perovskite films with different concentrations of PMAI. (b) XRD pattern of PMAI power and PMAI film by spin-coating 5000 rpm.

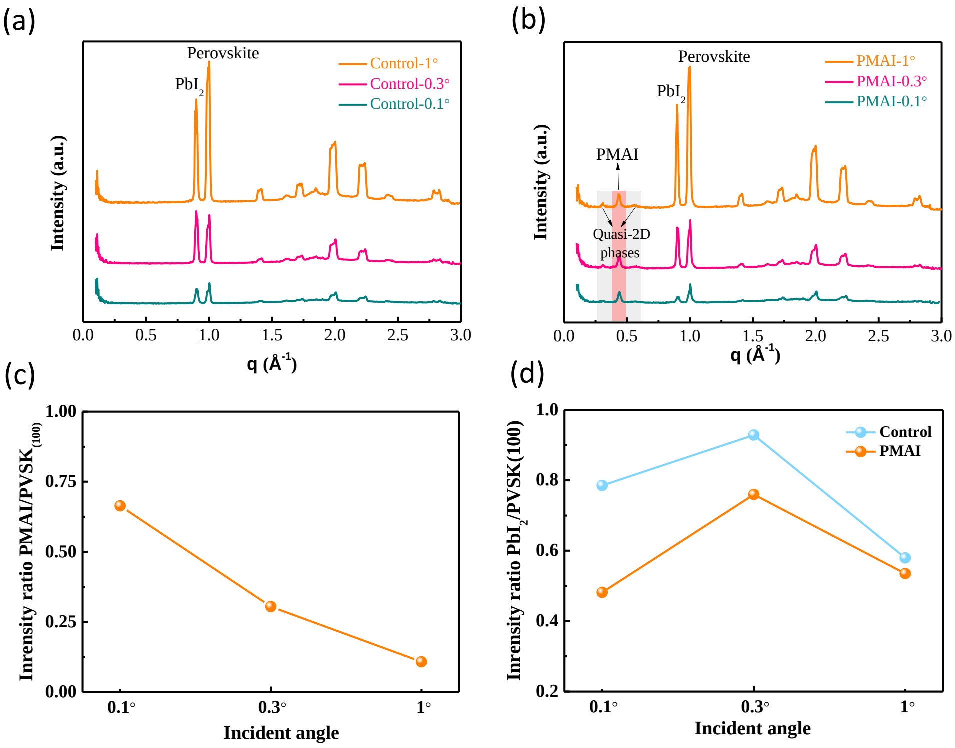

Figure S3. The GIWAXS intensity profiles of (a) the Control and (b) PMAI-treated perovskite films with three different incident angles. (c) The PMAI/PVSK (100) phase intensity ratio with different incidence angles. (d) The PbI_2/PVSK (100) phase intensity ratio with different incidence angles for the Control and PMAI-treated perovskite films.

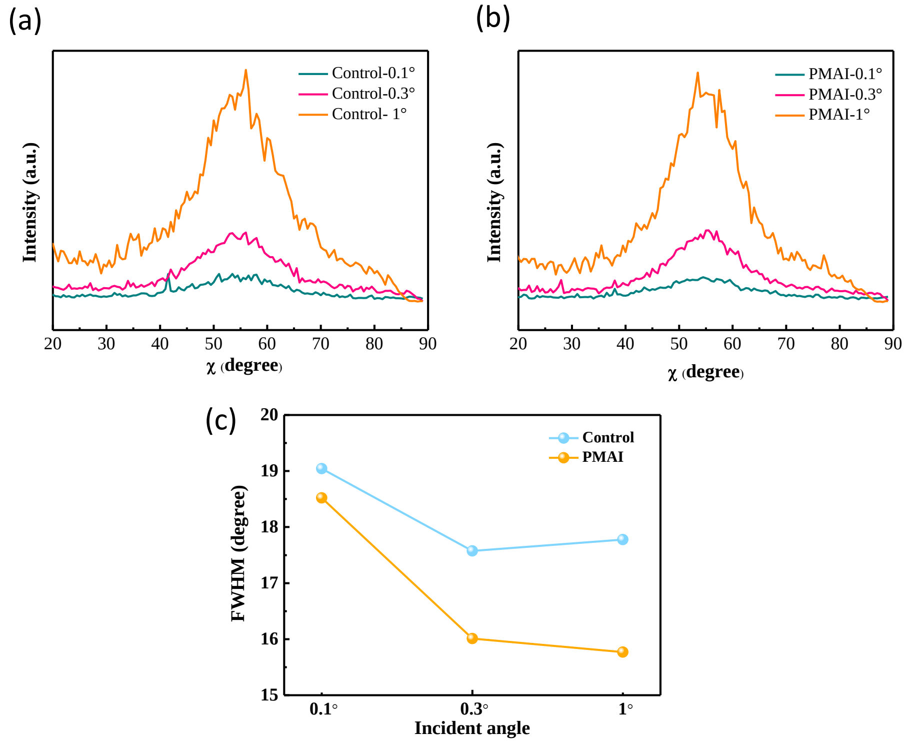

Figure S4. The polar intensity profiles along the ring at the range of 0.95-1.05 Å $^{-1}$ for (a) the Control and (b) PMAI-treated perovskite films. The full width at half maximum (FWHM) of the corresponding polar intensity profiles over azimuthal angle extracted from (100) diffraction for the Control and PMAI-treated perovskite films (c).

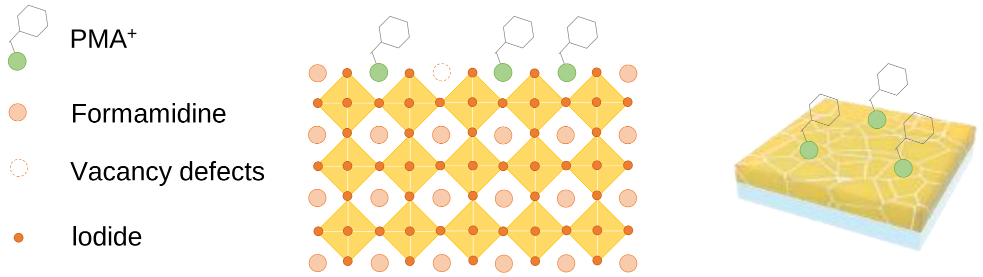

Figure S5. Diagram of potential passivation mechanism of the PMAI layer for the perovskite film.

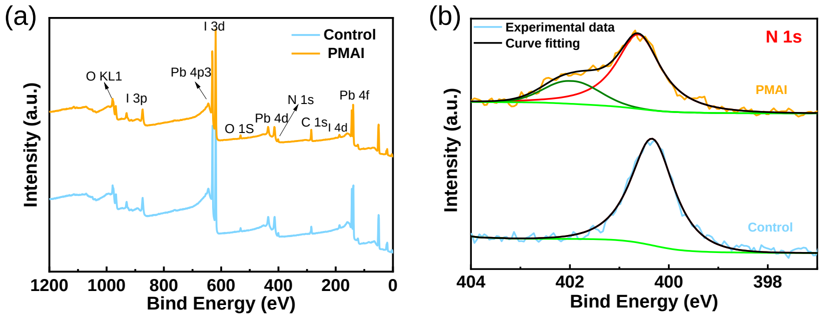

Figure S6. (a) Typical XPS survey spectrum of the perovskite films. (b) The N1s core-level energy spectra of the perovskite before and after PMAI treatment.

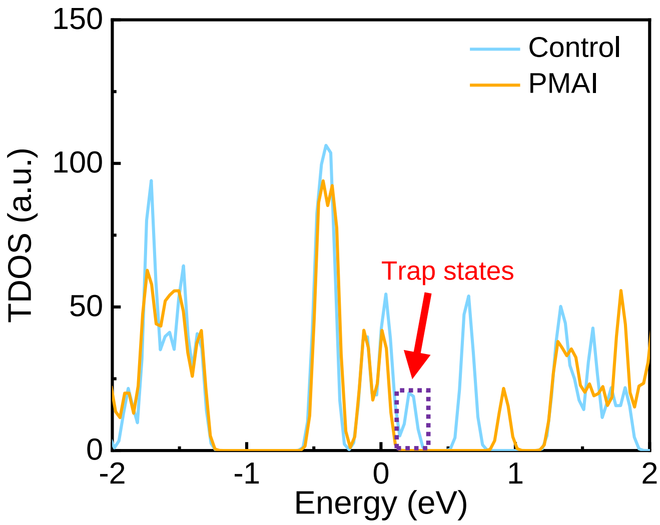

Figure S7. Density of states in $FAPbI_3$ (001) surface with and without PMAI.

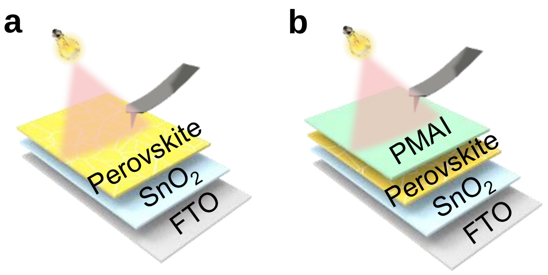

Figure S8. Schematic diagram of KPFM test device without (a) or with PMAI (b) layer.

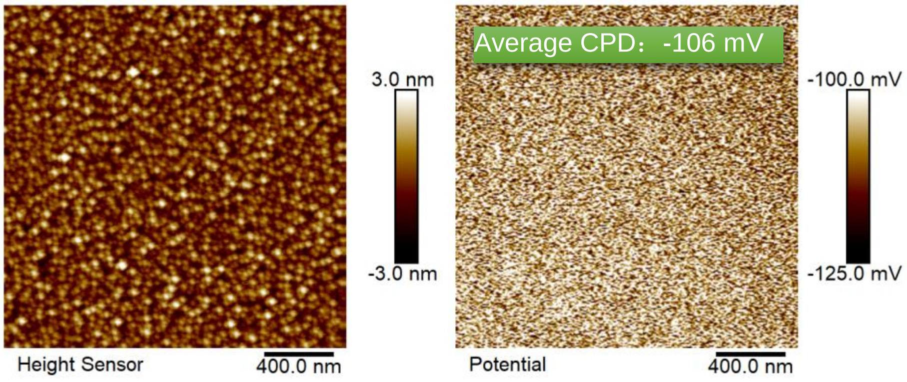

Figure S9. Calibration of probe potential in KPFM measurement using Au film. The average CPD of Au surface is -106 mV. The Work function of Au is 5.1 eV thus the probe tip potential is 4.994 V.

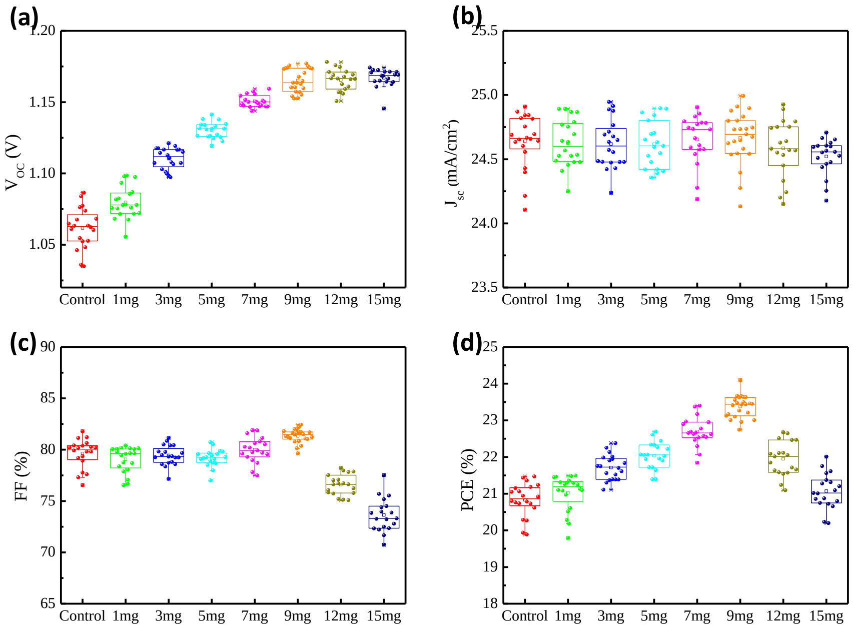

Figure S10. (a-d) Statistical distribution of photovoltaic parameters of different concentrations of PMAI.

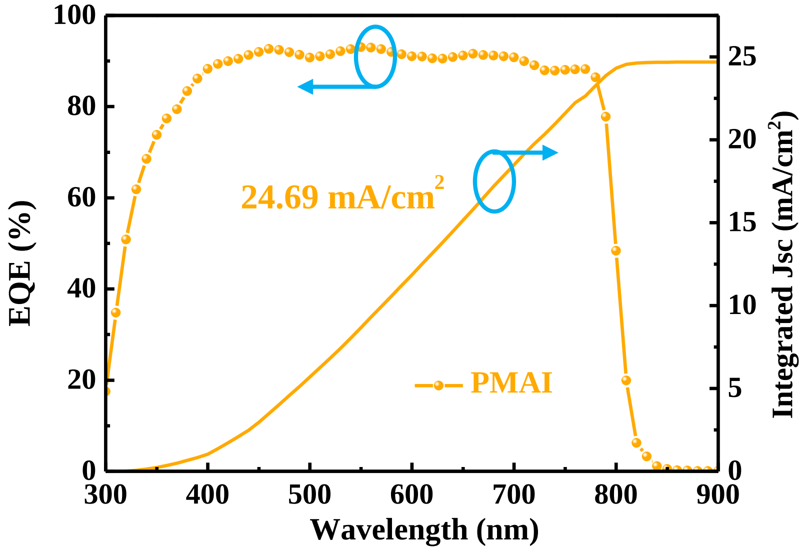

Figure S11. EQE and integrated J_{SC} of the cell treated with PMAI. The integrated J_{SC} from the EQE is 24.69 mA cm^{-2}. Note that the measured device sample here was from the same batch of the PCE-champion cell.

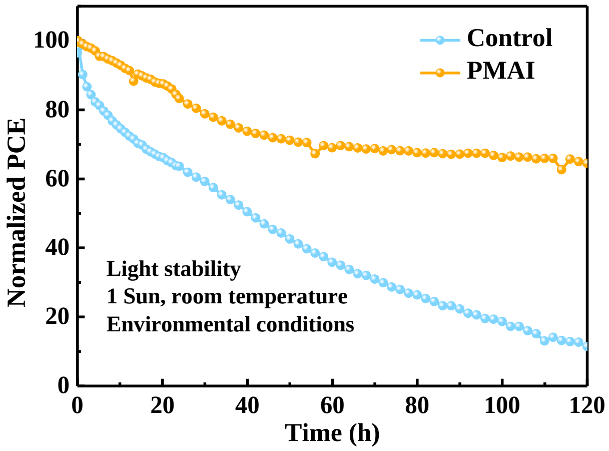

Figure S12. Light soaking stability under the illumination of 100 mW cm^{-2}.

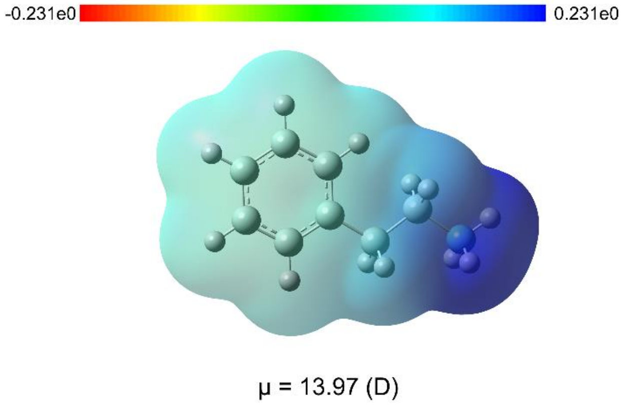

Figure S13. Calculated electronic density distributions with the dipole moment of PEA^{+}.

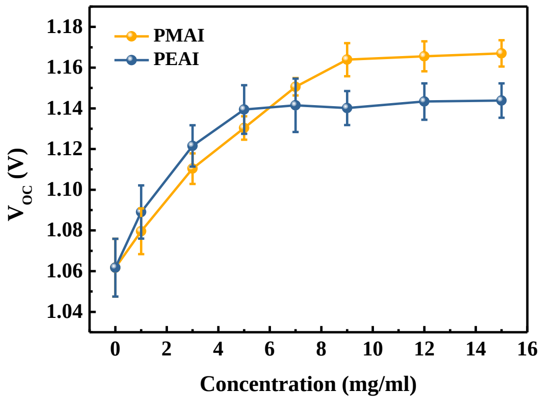

Figure S14. The average voltage of different concentrations of PMAI and PEAI.

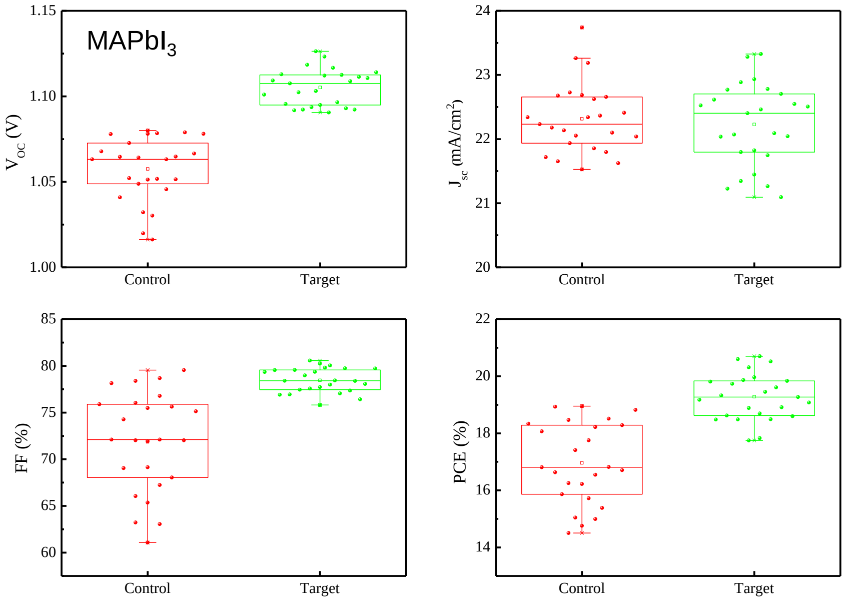

Figure S15. In the $MAPbI_3$ system, the statistical summary of photovoltaic parameters based on 25 devices before (Control) and after (Target) PMAI treatment.

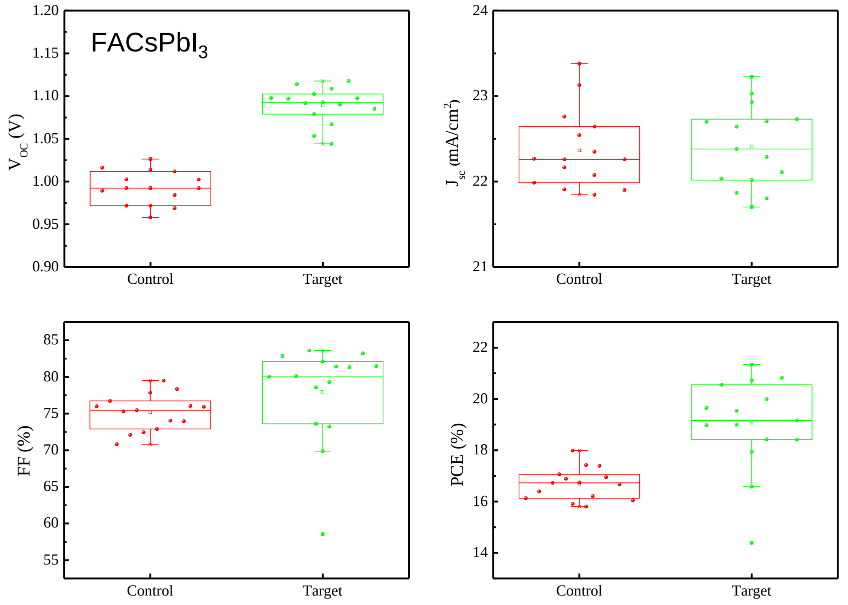

Figure S16. In the $FACsPbI_3$ system, the statistical summary of photovoltaic parameters based on 15 devices before (Control) and after (Target) PMAI treatment.

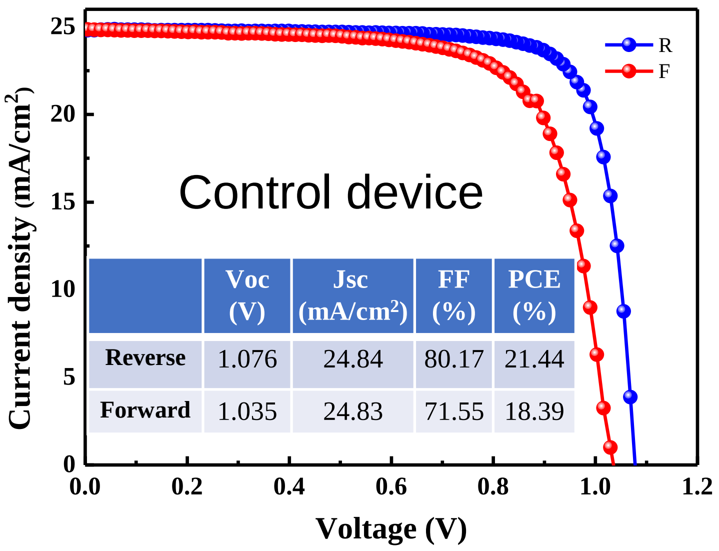

Figure S17. Current density–voltage (J–V) curves of the control device.

Table S1. Summary of fitting of the time-resolved photoluminescence (TRPL) of the perovskite films by exponential fitting.

Sample	A_1	τ_1 (μs)	A_2	τ_2 (μs)	τ_{Av} (μs)
Control	2016.9	0.946	3826.9	0.295	0.704
PMAI	3969.7	2.380	4745.2	0.697	1.944

Table S2. Summary of fitting impedance spectroscopy.

Sample	R_S(Ω)	R_C (Ω)	CPE
Control	10.6	619	8.85×10^{-8}
PMAI	9.1	354	12.7×10^{-8}

Table S3. Parameters applied in the wx-AMPS simulation. The simulation device structure was SnO_2/perovskite. For SnO_2, parameters were based on the defaults provided by the wx-AMPS software and the thickness was set to be 100 nm. For the perovskite layer, the thickness was 800 nm determined by SEM measurements. The wavelength-dependent absorption coefficients and bandgap were given by UV-Vis measurements. Effective densities of valence N_V and conduction N_C band states were based on reported values of $MAPbI_3$[2] and others were based on our previous work.[4]

Layer	SnO_2	Perovskite
Thickness[nm]	20.2	138.9
Permittivity	9	32
Band gap [eV]	3.6	1.53
Affinity [eV]	4	3.9
N_V [cm^{-3}]	2.2×10^{18}	1.9×10^{18}
N_C [cm^{-3}]	1.8×10^{19}	2.4×10^{18}
μ_n [$cm^2v^{-1}s^{-1}$]	100	0.5
μ_p [$cm^2v^{-1}s^{-1}$]	25	0.5
Surface recombination velocity S_0 [$cm\ s^{-1}$]	10^4	10^4
Surface recombination velocity S_L [$cm\ s^{-1}$]	10^4	10^4
Defect density [cm^{-3}]	1×10^{15}	1×10^{15}

References

[1] H. S. Duan, H. Zhou, Q. Chen, P. Sun, S. Luo, T. B. Song, B. Bob, Y. Yang, *Phys Chem Chem Phys* **2015**, 17, 112.
[2] T. Leijtens, G. E. Eperon, A. J. Barker, G. Grancini, W. Zhang, J. M. Ball, A. R. S. Kandada, H. J. Snaith, A. Petrozza, *Energy & Environmental Science* **2016**, 9, 3472.
[3] Q. Wang, C. Peng, L. Du, H. Li, W. Zhang, J. Xie, H. Qi, Y. Li, L. Tian, Y. Huang, *Adv. Mater. Interfaces* **2020**, 7, 1901866.
[4] P. Zeng, G. Feng, X. Cui, M. Liu, *The Journal of Physical Chemistry C* **2020**, 124, 6290.
